# Can digital X-ray radiogrammetry be an alternative for dual-energy X-ray absorptiometry in the diagnosis of secondary low bone quality in children?

**DOI:** 10.1007/s00431-019-03425-5

**Published:** 2019-07-27

**Authors:** Alex D. Leijten, Brieke Hampsink, Marcel Janssen, Willemijn M. Klein, Jos M. T. Draaisma

**Affiliations:** 1 Radboud Institute for Health Sciences, Radboudumc Amalia Children’s Hospital, Department of Pediatrics, Radboud University Medical Center, Nijmegen, The Netherlands; 2 Department of Radiology and Nuclear Medicine, Radboud University Medical Center, Nijmegen, The Netherlands

**Keywords:** Digital X-ray radiogrammetry, Dual-energy X-ray absorptiometry, Low bone quality, Osteoporosis

## Abstract

Bone quality in children is generally measured with dual-energy X-ray absorptiometry (DXA). Digital X-ray radiogrammetry (DXR) uses BoneXpert to measure cortical bone quality on hand radiographs. This prospective study compared DXR and DXA results in children with high probability of secondary low bone quality, defined as DXA of the lumbar spine (DXA_LS_) *Z*-score ≤ − 2.0. One hundred one children underwent both DXA and DXR assessment. DXA_LS_*Z*-scores were also adjusted for bone age. DXR *Z*-scores were compared with both DXA_LS_*Z*-scores, using Pearson correlations, Bland-Altman analysis, and sensitivity-specificity analysis. Mean bone age, DXR, and both DXA *Z*-scores were significantly impaired. Pearson correlation coefficients were significant between DXR *Z*-scores and both DXA_LS_*Z*-scores 0.507–0.564 (*p* < 0.001). Bland-Altman analysis showed a mean difference of 0.05–0.48 between DXR and both DXA *Z*-scores and showed more than 90% similarity for both DXA_LS_*Z*-scores ≤ − 2.0. DXR had a sensitivity of 67–71% and specificity of 77–83% compared to both DXA_LS_*Z*-scores.

*Conclusion*: DXR correlates well with as well DXA_LS_ as bone age-adjusted DXA_LS_*Z*-scores and shows good agreement with as well DXA_LS_ as bone age-adjusted DXA_LS_*Z*-scores ≤ − 2.0. DXR shows best results when compared with DXA_LS_*Z*-scores.

**Table:** 

**What is Known:** *• Digital X-ray radiogrammetry (DXR) may correlate well with dual-energy X-ray absorptiometry (DXA) in pediatric, adolescent, and adult patients.* *• DXR is a feasible method for assessment of bone quality in children.*	
**What is New:** *• This is the first prospective study in children with suspected secondary low bone quality that illustrates correlation between DXR and bone age-adjusted DXA Z-scores and that shows good agreement between DXR and DXA as bone age-adjusted DXA Z-scores ≤ −2.0.* *• Our results suggest DXR to be a good alternative for DXA for determining low bone quality.*

**What is Known:**

*• Digital X-ray radiogrammetry (DXR) may correlate well with dual-energy X-ray absorptiometry (DXA) in pediatric, adolescent, and adult patients.*

*• DXR is a feasible method for assessment of bone quality in children.*

**What is New:**

*• This is the first prospective study in children with suspected secondary low bone quality that illustrates correlation between DXR and bone age-adjusted DXA Z-scores and that shows good agreement between DXR and DXA as bone age-adjusted DXA Z-scores ≤ −2.0.*

*• Our results suggest DXR to be a good alternative for DXA for determining low bone quality.*

## Introduction

Osteoporosis is a multifactorial skeletal disorder resulting in bone fragility and is associated with fractures, morbidity, and mortality [22]. Pediatric osteoporosis is generally categorized in primary and secondary forms. Secondary osteoporosis and thus also secondary low bone quality in children is caused by systemic disease, their treatment, or indirect effects of systemic disease, such as immobilization, reduced time spent outdoor, and poor nutrition, or a combination of these factors [28].

Osteoporosis in children is defined differently than in adults, because bone mass (bone mineral density, BMD) varies greatly with age. Therefore, bone densitometry uses pediatric *Z*-scores that refer to an age-appropriate cohort of healthy children and adolescents, instead of adult *T*-scores [1, 26]. *Z*-scores ≤ − 2.0 are defined as low bone density or mass for age [14]. According to the 2013 International Society of Clinical Densitometry, pediatric osteoporosis is defined as low bone density for age in combination with a clinically significant fracture history, i.e., ≥ 2 long bone fractures before age of 10 or ≥ 3 long bone fractures before age of 19, or the presence of one or more vertebral compression fractures occurring without major trauma or local disease [2].

Peak bone mass is a strong predictor of fracture risk and osteoporosis in adulthood. Because 90% of peak bone mass is acquired by the age of 18, any (in)direct effects of pediatric disease influence bone status in both childhood and adulthood [22, 28]. Incidence records of secondary low bone mineral density (BMD: an equivalent of bone mass) vary; however, the incidence of low BMD in non-ambulatory children with cerebral palsy is reported up to 97% [7, 11]. Children with limited ambulation typically have low BMD and many will sustain fractures [8].

Dual-energy X-ray absorptiometry (DXA) is the golden standard for bone quality measurement in children as well as adults, due to precision, reproducibility, and availability of normative data [14, 19]. DXA measurements give information about BMD of the site studied. Different skeletal sites are described for BMD measurement in children. DXA of the lumbar spine L1–L4 (DXA_LS_) is a recommended site and is superior to DXA of the femur or fore arm [14]. Nevertheless, DXA has its limitations. Disrupting factors as movement during measurement, metallic implants, contractures, and sometimes even scoliosis can cause results to be non-interpretable. In addition, the *Z*-scores are based on calendar age and do not take bone age into account, hence, may provide inaccurate findings [8, 12, 13]. Finally, DXA provides measurement of areal BMD (g/cm^2^), rather than volumetric density (g/cm^3^), which may result in underestimation of BMD in children with small or narrow bones and overestimation of BMD in children with tall stature [1, 8]. These limitations feel the need for alternative methods.

The clinically available digital X-ray radiogrammetry (DXR) of the hand seems a feasible alternative. Using a web-based software like BoneXpert, it can assess both bone age and bone quality, expressed as bone health index (BHI), a measure of cortical thickness and mineralization, which may result in an accurate representation of bone quality. The BHI reference values are gender and bone age specific. DXR is less stressful compared to DXA, easy to obtain, and often does not involve additional exposure to ionizing radiation, since hand radiographs for the assessment of bone age are regularly obtained in disabled children that are prone to low bone quality [13, 19, 27].

Several studies in pediatric, adolescent, and adult populations showed that bone quality measured by DXR may correlate well with DXA measurements [3, 17–19, 24]. Other studies mentioned sensitivity and specificity of DXR compared to DXA of the lumbar spine and/or total body bone mineral density as the “golden standard,” varying from 40–90 to 79–93%, respectively [15, 16]. However, these studies were performed in specific (i.e., children with juvenile idiopathic arthritis or intestinal failure) and small (*n* = 24–35) pediatric populations [15, 16]. This raises the question whether DXR is a viable alternative for DXA for bone health assessment in children with high probability of secondary low bone quality, which has not yet been proven.

This study compares the measurements of DXR and DXA performed in children with high probability of secondary low bone quality, thus determining the diagnostic accuracy of DXR as a method for determining bone health in these children.

## Materials and methods

### Patients

Patients were sampled from the outpatient clinic of the Radboudumc Amalia Children’s hospital, Nijmegen, the Netherlands. We selected patients visiting the outpatient clinic between July 2016 and January 2019 with a high probability of low bone density. Patients were included if both DXA and conventional radiographs of the non-dominant hand were performed. If possible, both tests were performed on the same day or else at least within 3 months. We selected patients older than 3 years of age, the lower limit of *Z*-scores available for BMD of the lumbar spine L1–L4 (BMD_LS_), and younger than the reliability range of the DXR-measurements, i.e., 19 years for boys and 18 years for girls [25]. Patients were excluded if either BMD_LS_ or BHI could not be assessed. This study has been approved by the Medical Ethics Committee at Radboudumc Nijmegen (file number 2016-2946). Informed consent was acquired from all parents and, when appropriate, patients aged 12 years or older.

### Study procedures

This was a prospectively planned cross-sectional diagnostic comparative study. All inclusions and measurements took place in the Radboudumc Amalia Children’s hospital, Nijmegen, the Netherlands. We performed both DXR and DXA scan in children with high probability of low bone quality due to (in)direct effects of systemic disease or their treatment.

### Digital X-ray radiogrammetry

Conventional radiographs of the non-dominant hand, usually the left, were taken. DXR was processed using the BoneXpert (BoneXpert, Version 2.4.5.1, Visiana, Holte, Denmark) software. The BoneXpert software calculates the BHI based on the cortical thickness (*T*), width (*W*), and length (*L*) of the metacarpals, expressed in the following formula: BHI = *πT*(1 − *T*/*W*)/(*LW*)^0.33^. It automatically compares the BHI to a Danish and Dutch reference population with same sex and bone age, expressed as a *Z*-score [24]. The bone age calculation in BoneXpert is based on Greulich and Pyle [25].

### Dual-energy X-ray absorptiometry

A Hologic Discovery A S/N 85606 DXA scanner was used to measure BMD expressed as g/cm^2^. DXA_LS_ is a recommended skeletal site for BMD assessment in children, due to speed and accuracy [14]. Therefore, patients in our study had to undergo at least one successful assessment of BMD_LS_. BMD-measurements were compared to normative values based on ethnicity, age, and sex, as provided by the manufacturer, expressed as *Z*-scores [9, 30]. Ultimately, BMD_LS_*Z*-scores were corrected for bone age, using the results from the BoneXpert software.

### Statistical analysis

The estimated sample size was based on a power calculation [6]. As the likelihood ratio combines the sensitivity and specificity of a test, we used this index to calculate the sample size. With a sensitivity and specificity of 90%, the calculated minimal required sample size for a pre-determined value of positive likelihood ratio of 5.0 and a negative likelihood ratio of 0.20 (within a 95% confidence level) was 101. All statistical analyses were performed using SPSS Version 25.0 (IBM, Armonk, New York). Patient characteristics were documented as continuous values, mean ± standard deviation (SD) when normally distributed or median and range when not normally distributed. Comparisons of bone age and BMD measurements to a reference population were performed with one sample *t* tests. To evaluate the possible impact of ambulatory status on bone age and BMD measurements, independent sample *t* tests were performed.

All analyses compared BHI *Z*-scores with BMD_LS_ and bone age-adjusted BMD_LS_*Z*-scores. DXA measurements were used as the golden standard in all analyses. Correlations between DXR and DXA *Z*-scores were assessed with Pearson correlation coefficients. Subgroup analysis for ambulatory status was performed with Fisher *r*-to-*z* transformation as described by Snedecor et al. [21]. Agreement between DXR and DXA *Z*-scores was assessed with Bland-Altman analysis, 95% confidence interval limits of agreement were calculated and linear regression was performed to rule out proportional bias. Coherence between both Bland-Altman analyses was calculated with correlation coefficients and paired sample *t* tests. Percentage similarity was calculated to show relative agreement between BMD_LS_ and bone age-adjusted BMD_LS_*Z*-scores and BHI *Z*-scores according to the following formula: [((*A*/*B*)/2)/*A*] *×* 100%; *A* represents the BMD_LS_ and bone age-adjusted BMD_LS_*Z*-scores and *B* the BHI *Z*-scores [20]. To evaluate impact of bone age-adjusted BMD_LS_*Z*-scores, paired sample *t* tests were performed.

To determine if DXR can correctly diagnose a low bone density in patients with a DXA *Z*-score ≤ − 2.0, a sensitivity-specificity analysis was conducted. Subgroup analyses were performed for full ambulatory (gross motor function classification system ≤ 2) and (outdoor) non-ambulatory (gross motor function classification system ≥ 3) patients. *p* values < 0.05 were considered statistically significant.

## Results

### Patients

**Fig. 1:**
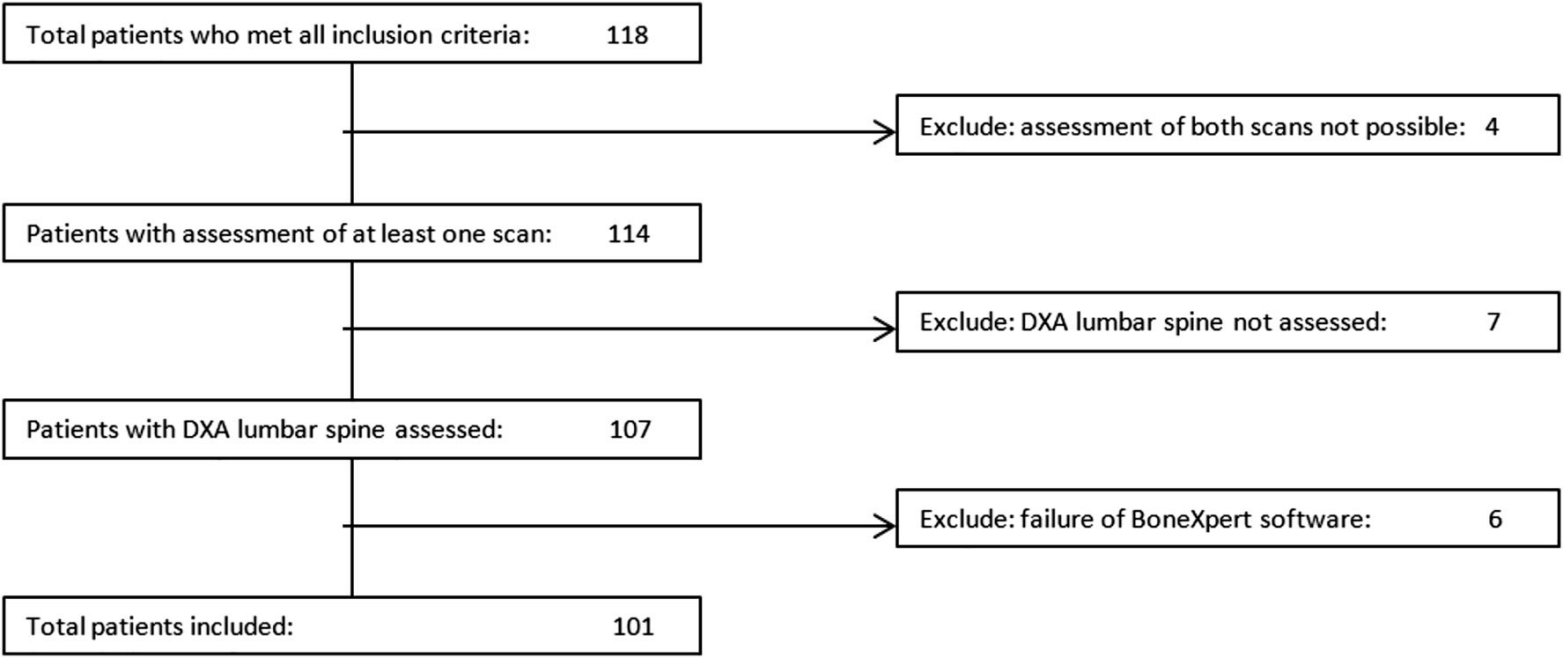
Flowchart of patient exclusion. DXA, dual-energy X-ray absorptiometry

One hundred eighteen patients were eligible for the study, of whom 17 could not be included due to unavailable test results (Fig. 1). The median time interval between the DXA scan and hand radiograph was 0 day, with only three outliers > 50 days (55, 86, and 91 days).

Patient characteristics of the included 101 patients are shown in Table 1. Sixty-three (62%) patients were fully ambulatory and 38 (38%) were (outdoor) non-ambulatory. Twenty-seven out of 38 (71%) non-ambulatory patients were severely disabled (gross motor function classification system ≥ 4). Mean bone age *Z*-score was significantly lower than that of healthy peers with the same age (mean *Z*-score − 0.5, *p* = 0.001). Mean bone age *Z*-score of ambulatory patients did not differ from non-ambulatory patients (*p* = 0.76).

**Table 1:** Patient characteristics and demographics

	*n* = 101
Male gender, *n* (%)	55 (55)
Main diagnoses, *n*	
Scoliosis	36
Epilepsy	34
Neurological disorder, non-epileptic	18
Genetic mutation, non-epileptic	8
Syndromic disorder	16
Pulmonary disease	10
Gastrointestinal disease	4
Childhood cancer	4
Fractures with unknown cause	4
Patients with ≥ 1 diagnoses, *n* (%)	29 (29)
Ambulatory status, *n* (%)	
Full ambulatory (GMFCS ≤ 2)	63 (62)
(Outdoor) non-ambulatory (GMFCS ≥ 3)	38 (38)
Severely disabled (GMFCS ≥ 4)	27 (71)
Time between DXA and DXR in days, median (range)	0 (0–91)
Age at DXR in years, mean (SD)	11.7 (± 3.8)
Bone age at DXR in years, mean (SD)	10.9 (± 3.7)
*Z*-score bone age, mean (SD)	− 0.5 (± 1.4)*

*GMFCS* gross motor functioning classification system, *DXA* dual-energy X-ray absorptiometry, *DXR* digital X-ray radiogrammetry, *SD* standard deviation

*p value = 0.001 on one sample *t* test with test value 0

### Feasibility

It was possible to assess the BHI in 108/118 (92%) individuals (Fig. 1). All 10 unsuccessful measurements were in severely disabled children and were due to flexion/contractures of the hand (50%), anatomical bone deformities (30%), or failure to assess bone age (20%). Assessment of BMD_LS_ was not possible in 11/118 (9%) patients. Nine out of 11 (82%) patients that failed BMD_LS_ assessment were severely disabled. The most common cause of inability to analyze BMD_LS_ in this group was incapability to lie still (50%). In the two full ambulatory patients, measurement was impossible due to presence of osteosynthesis material.

In 4 children, it was not possible to determine both BMD_LS_ as well as BHI. All of these children were severely disabled. In this group, BHI assessment failed due to flexion/contractures of the hand (3 children) and anatomical bone deformities (1 child), and BMD_LS_ assessment was not possible because of inability to lie still (3 children) and spasms (1 child).

### Bone health assessment

A total of 101 combinations of BMD_LS_ and BHI measurements were obtained. Mean BMD_LS_, BHI *Z*-scores, and mean bone age-adjusted BMD_LS_*Z*-scores were significantly lower (*p* < 0.001) than a reference population with *Z*-score 0 (Table 2) [9, 24, 30]. Compared to the fully ambulatory patients, non-ambulatory children had significant lower mean BMD_LS_ (*p* < 0.001), BHI (*p* < 0.001), and mean bone age-adjusted BMD_LS_ (*p* < 0.001) *Z*-scores.

**Table 2:** Bone health assessment of patients divided into three categories

	Mean BMD_LS_*Z*-score	Mean BHI *Z*-score	Mean bone age-adjusted BMD_LS_*Z*-score
All patients (*n* = 101)	− 1.3 (± 1.8)*	− 1.3 (± 1.6)*	− 0.8 (± 1.5)*
Full ambulatory (*n* = 63)	− 0.7 (± 1.5)	− 0.9 (± 1.4)	− 0.9 (± 1.4)
(Outdoor) non-ambulatory (*n* = 38)	− 2.2 (± 1.8)**	− 2.0 (± 1.6)**	− 1.7 (± 1.3)**

*DXR* digital X-ray radiogrammetry, *BMD*_*LS*_ lumbar spine bone mineral density, *BHI* bone health index, *SD* standard deviation

*p value < 0.001 on one sample *t* test with test value 0

**Significant difference compared to full ambulatory patients (*p* < 0.001)

### Correlation between DXA and DXR

**Fig. 2:**
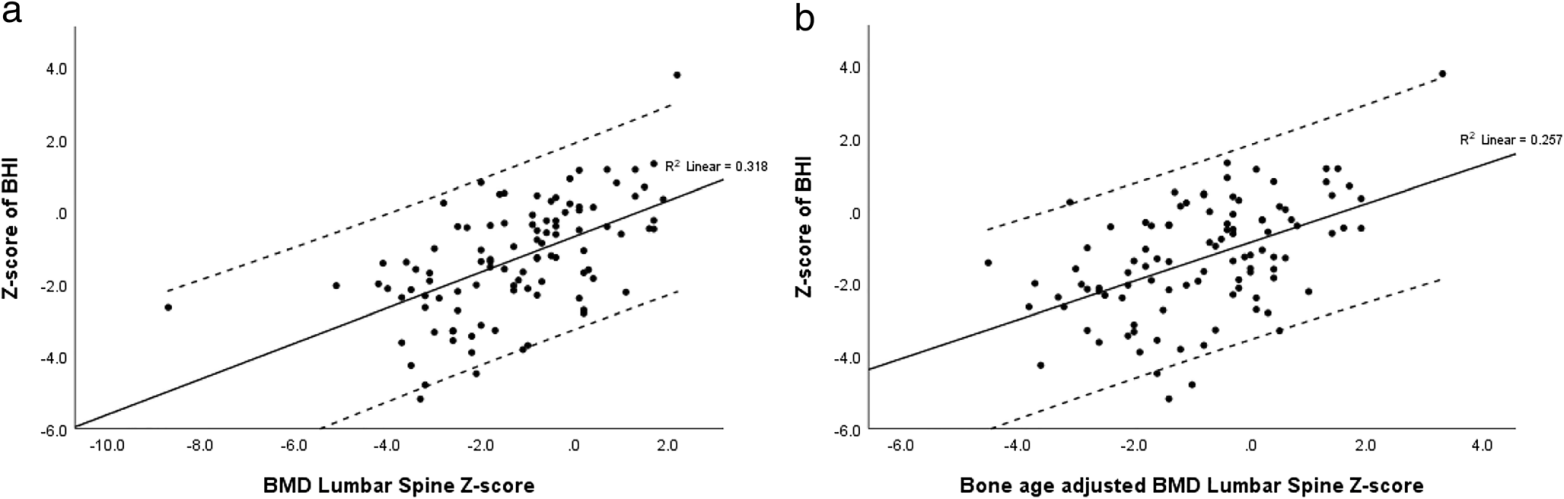
Correlation between DXR and DXA measurements for BMD lumbar spine *Z*-scores and BHI *Z*-scores (**a**) and bone age-adjusted BMD lumbar spine *Z*-scores and BHI *Z*-scores (**b**). BMD, bone mineral density; BHI, bone health index

All BHI *Z*-scores and DXA as well as bone age-adjusted DXA *Z*-scores were positively and significantly correlated (BHI *Z*-score and BMD_LS_*Z*-score, 0.564, *p* < 0.001; BHI *Z*-score and bone adjusted BMD_LS_*Z*-score, 0.507, *p* < 0.001). All correlation coefficients were lower for the non-ambulatory group compared to the full ambulatory group (data not shown), but these differences were not statistically significant using the Fisher *r*-to-*z* transformation (Fig. 2).

### Comparison between DXA and DXR

#### Agreement between DXA and DXR

**Fig. 3:**
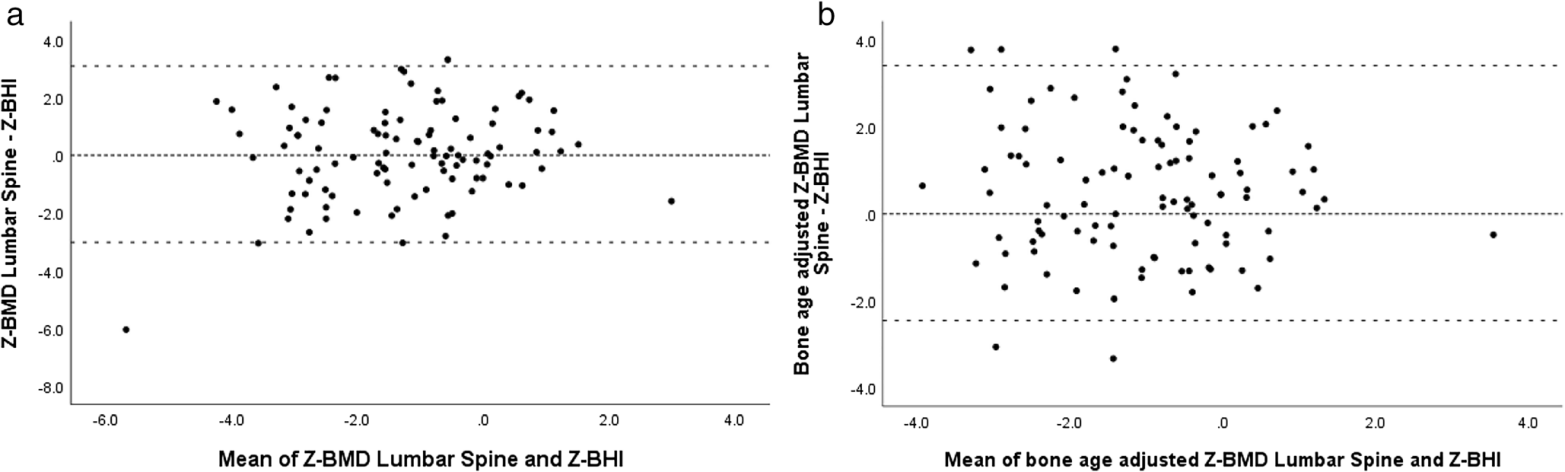
Bland-Altman graphs illustrating similarity between absolute scores of BMD lumbar spine and BHI (**a**) *Z*-scores and bone age-adjusted BMD lumbar spine and BHI *Z*-scores (**b**). The *X*-axis indicates the mean of the two methods (DXA and DXR) and the *Y*-axis shows the difference. The small dotted line represents a difference of 0 and the large dotted line illustrates the 95% limits of agreement. Z-BMD, bone mineral density *Z*-scores; Z-BHI, bone health index *Z*-score; DXA, dual-energy X-ray absorptiometry; DXR, digital X-ray radiogrammetry

Correlation can describe a linear relationship between two methods of measurement, but does not necessarily imply agreement [4]. Therefore, we conducted Bland-Altman analysis, shown in the Bland-Altman plots (Fig. 3). BMD_LS_ and BHI *Z*-scores showed a non-significant mean bias of 0.05. Bone age-adjusted BMD_LS_ and BHI *Z*-scores showed a significant mean bias of 0.48 (*p* = 0.002). Linear regression did not demonstrate proportional bias for both comparisons. Differences between BHI *Z*-scores and BMD_LS_*Z*-scores, and BHI and bone age-adjusted BMD_LS_*Z*-scores, showed strong correlation (*r* = 0.821; *p* < 0.001).

**Fig. 4:**
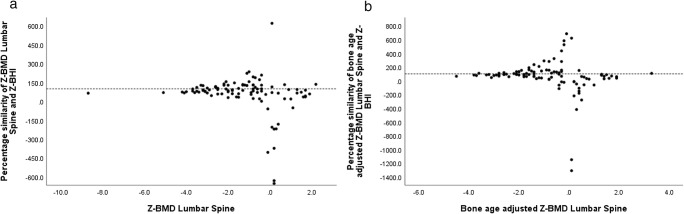
Bland-Altman graphs illustrating the percentage similarity. The *X*-axis shows the absolute value of BMD lumbar spine (**a**) and bone age-adjusted BMD lumbar spine *Z*-scores (**b**); the *Y*-axis indicates the percentage similarity between BMD lumbar spine and BHI *Z*-scores (**a**), and bone age-adjusted BMD lumbar spine and BHI *Z*-scores (**b**). The dotted line represents a similarity of 100%. Z-BMD, bone mineral density *Z*-scores; Z-BHI, bone health index *Z*-score

To show relative agreement, we plotted percentage similarity between (BA-)BMD_LS_*Z*-scores and BHI *Z*-scores (Fig. 4). BMD_LS_ and BHI *Z*-scores showed a mean similarity of 47.9% and bone age-adjusted BMD_LS_ and BHI *Z*-scores a mean similarity of 66.7%. BHI *Z*-scores showed a percentage similarity of 90.9% with BMD_LS_*Z*-scores for BMD_LS_*Z*-scores ≤ − 2.0, which was significant different compared to BMD_LS_*Z*-scores > − 2.0 (*p* = 0.029). In addition, BHI Z-scores demonstrated a percentage similarity of 92.4% with bone age-adjusted BMD_LS_*Z*-scores for *Z*-scores ≤ − 2.0, which was not significant different compared to bone age-adjusted BMD_LS_*Z*-scores > − 2.0 (*p* = 0.304).

#### Classification of bone health by DXR compared to DXA

DXR had a sensitivity of 67% and a specificity of 83% for low bone mineral density (BMD_LS_*Z*-score ≤ − 2.0). When BMD_LS_ results were corrected for bone age, sensitivity, and specificity were respectively 71% and 77%.

Subgroup analysis calculated distinguishing features for full ambulatory and non-ambulatory children. In full ambulatory children, DXR had a sensitivity of 53% (BMD_LS_*Z*-score) and 67% (bone age-adjusted BMD_LS_*Z*-score). Specificity was 88% (BMD_LS_*Z*-score) and 85% (bone age-adjusted BMD_LS_*Z*-score). Sensitivity and specificity were respectively 76% and 71% (BMD_LS_*Z*-score) and 73% and 57% (bone age-adjusted BMD_LS_*Z*-score) in non-ambulatory children.

## Discussion

Our results show that DXR may be a promising alternative for measuring bone health in children with high probability of secondary low bone quality. BHI has a significant and positive correlation with all DXA measurements. Agreement between BHI and as well BMD_LS_ as bone age-adjusted BMD_LS_ is high, especially for *Z*-scores ≤ − 2.0. BHI Z-scores show best diagnostic performance when compared with BMD_LS_*Z*-scores without correction for bone age.

To our knowledge, this is the first prospectively planned cross-sectional study comparing DXR and DXA in a diverse group of children with high probability of secondary low bone quality and the first study to compare DXA scores corrected for bone age. We used DXR with automated calculation of bone age and bone quality using the BoneXpert software. The advantages of DXR over DXA are its low burden on patients, no influence of soft tissue thickness on bone quality calculations, and since many children with suspicion of low bone quality have an indication for a hand radiograph to analyze bone age, the extra software postprocessing does not involve additional exposure to ionizing radiation [10, 13, 27]. Additionally, conventional radiographs of the hand can be performed in any hospital in the Netherlands, in contrary to DXA [13].

Previous studies compared DXR and DXA measurements in different groups of children; however, only a few compared DXA *Z*-scores with DXR. The correlation coefficients between BMD_LS_ and BHI *Z*-scores in our study were higher than those found in children with juvenile idiopathic arthritis or children with suspected secondary low bone mineral density [16, 19]. A study in children with inflammatory bowel disease showed comparable correlation coefficients between BMD_LS_ and BHI *Z*-scores [4]. Neelis et al. showed slightly higher correlation coefficients in children with intestinal failure [15]. That study also attempted to show agreement between BMD_LS_ and BHI. Limits of agreement were comparable with our study; however, their variability was non-consistent, most likely due to their small number of participants. Sensitivity and specificity of DXR compared to DXA_LS_ were similar [15].

Unfortunately, DXR assessment was not possible in 10 of 118 (8%) of eligible patients, due to overprojection of metacarpals, anatomical bone deformities, and missing bone age. This percentage is higher than in previous studies that showed 1.4 to 7.6% [15, 19, 23]. However, in our study DXR only failed in severely disabled patients. If ambulatory status is taken into account, DXR had a feasibility of 100% in full ambulatory patients and was successful in 27/37 (73%) severely disabled patients. This is better than the 63.2% reported in a feasibility study in severely disabled children [13].

We investigated correlation, agreement, and distinguishing features of DXR compared to DXA_LS_, since DXA_LS_ is a recommended skeletal site for BMD measurement in children and is superior to DXA of the femur or fore arm [14]. BHI and BMD_LS_*Z*-scores correlated well and showed moderate to good agreement. Percentage similarity showed good agreement for *Z*-scores ≤ − 2.0. Nonetheless, in 27 (27%) patients BHI and BMD_LS_*Z*-scores differed greatly (> 2 *Z*-scores). This difference was caused by a large discrepancy between bone and calendar age in 9/27 (33%) individuals. In 16 of the 18 (89%) remaining patients, BMD_LS_ was higher than the BHI *Z*-score, probably because DXR is described to be more sensitive to irregularities than DXA [15]. Another possible explanation is that DXR is sensitive to a decrease in the amount of bone tissue, but unsuitable for bone mineralization defects, especially when affecting the cortical bone [19, 24]. Certain syndromic disorders and genetic disorder that are known for altered bone mineralization may cause differences between DXA and DXR. Furthermore, DXR only measures cortical bone [24], and therefore, disease and/or medication that alter trabecular bone may create differences in BMD measurements by DXR and DXA. However, for clinical practice, it is most important to determine low bone quality (*Z*-scores ≤ − 2.0). Our results showed good agreement between BHI and BMD_LS_ and bone age-adjusted BMD_LS_ for *Z*-scores ≤ − 2.0. Additionally, in this series with high prevalence of children with low bone quality, DXR showed good negative predictive value of 82% and 89% when compared to BMD_LS_ and bone age-adjusted BMD_LS_*Z*-scores. These values were even higher for ambulatory patients (BMD_LS_*Z*-score, 86%; bone age-adjusted BMD_LS_*Z*-scores, 94%), most likely because immobility induces altered bone geometry, i.e., thinner cortices and reduced cortical diameters [5], resulting in lower BHI values for non-ambulatory patients.

In the present study, bone density was significantly impaired. As shown in Table 2, it is presumable that ambulatory status had a large contribution to the impaired mineral bone density in all patients. Ambulatory status did not influence bone age. Bone age was significantly impaired compared to a healthy cohort; hence, we adjusted the DXA *Z*-scores for bone age and compared these with the BHI *Z*-scores, as was suggested earlier [15].

Bone age is associated with pubertal maturation and could therefore be an advantage [29]. Unexpectedly, DXA scores adjusted for bone age were slightly worse correlated with BHI *Z*-scores than DXA Z-scores. In addition, comparison of bone age-adjusted DXA_LS_*Z*-scores with BHI *Z*-scores showed a significant mean bias of 0.48. Although this mean bias differed significantly from the mean difference between DXA_LS_*Z*-scores with BHI *Z*-scores, differences from the Bland-Altman analyses showed a very strong positive correlation. Similarity percentage between BHI and bone age-adjusted DXA_LS_*Z*-scores were comparable for *Z*-scores ≤ − 2. We hypothesize that these results are most likely due to lack of reference range data for bone age-adjusted DXA_LS_ scores [5]. In the present study, we used the same reference database for as well the DXA_LS_ as the bone age-adjusted DXA_LS_*Z*-scores. It is probable that normal distribution between bone age and BMD diverge; therefore, bone age-adjusted DXA_LS_*Z*-scores can differ from measured DXA_LS_*Z*-scores. In addition, the bone age was significantly impaired in our cohort, resulting in higher bone age-adjusted DXA_LS_*Z*-scores than measured DXA_LS_*Z*-scores. Moreover, it is likely that differences between bone age-adjusted DXA_LS_ and measured DXA_LS_*Z*-scores tend to be more pronounced when there is a larger difference between bone age and calendar age, as for our population.

Strengths of our study include its prospectively planned cross-sectional design. The amount of patients included is substantially larger than in previous studies [10, 15–17]. In addition, our patient group is heterogeneous in both ambulatory status and medical conditions that could lead to secondary low BMD. Therefore, our study population is representative for a large amount of patients that might need bone quality assessment.

Further, the median time interval between DXA and hand radiographs was 0 day. This makes our comparison more reliable, especially for the bone age-adjusted DXA *Z*-scores, compared to studies where the time interval was up to 8 months [19].

Some limitations of our study should be addressed. Subgroup analyses were difficult to interpret, because the non-ambulatory group was smaller than the full ambulatory patient group. Also, since we only included patients who underwent both DXA and DXR, there may be a selection bias. Some parents did not want to make an additional DXR. In addition, clinicians’ interpretation of a high probability of low bone mineral density could vary, and therefore, there could be a selection bias.

In conclusion, DXR and DXA measurements correlate well. BHI and as well BMD_LS_ as bone age-adjusted BMD_LS_*Z*-scores show good agreement for *Z*-scores ≤ 2.0, especially the comparison of BHI and BMD_LS_*Z*-scores. Our results suggest DXR to be a promising alternative for DXA for determining low bone quality in children with suspected secondary low bone quality or osteoporosis. Future research should include gathering of reference data for bone age-adjusted DXA *Z*-scores, the value of DXR in predicting future fracture risk, and the value of DXR in measuring the therapeutic effects of different interventions. For these last two reasons, prospective, longitudinal studies are required.

## Abbreviations

BHI: Bone health index
BMD: Bone mineral density
BMD_LS_: Bone mineral density of the lumbar spine
DXA: Dual energy X-ray absorptiometry
DXA_LS_: Dual-energy X-ray absorptiometry of the lumbar spine
DXR: Digital X-ray radiogrammetry

## References

[1] Bachrach LK, Gordon CM, Section On E (2016) Bone densitometry in children and adolescents. Pediatrics 138(4). 10.1542/peds.2016-2398 10.1542/peds.2016-2398 27669735

[2] Bishop N, Arundel P, Clark E, Dimitri P, Farr J, Jones G, Makitie O, Munns CF, Shaw N, International Society of Clinical D (2014). Fracture prediction and the definition of osteoporosis in children and adolescents: the ISCD 2013 Pediatric Official Positions. J Clin Densitom.

[3] Dhainaut A, Rohde GE, Syversen U, Johnsen V, Haugeberg G (2010). The ability of hand digital X-ray radiogrammetry to identify middle-aged and elderly women with reduced bone density, as assessed by femoral neck dual-energy X-ray absorptiometry. J Clin Densitom.

[4] Giavarina D (2015). Understanding Bland Altman analysis. Biochem Med (Zagreb).

[5] Grover M, Bachrach LK (2017). Osteoporosis in children with chronic illnesses: diagnosis, monitoring, and treatment. Curr Osteoporos Rep.

[6] Hajian-Tilaki K (2014). Sample size estimation in diagnostic test studies of biomedical informatics. J Biomed Inform.

[7] Henderson RC, Lark RK, Gurka MJ, Worley G, Fung EB, Conaway M, Stallings VA, Stevenson RD (2002). Bone density and metabolism in children and adolescents with moderate to severe cerebral palsy. Am Acad Pediatr.

[8] Henderson RC, Berglund LM, May R, Zemel BS, Grossberg RI, Johnson J, Plotkin H, Stevenson RD, Szalay E, Wong B, Kecskemethy HH, Harcke HT (2010). The relationship between fractures and DXA measures of BMD in the distal femur of children and adolescents with cerebral palsy or muscular dystrophy. J Bone Miner Res.

[9] Kelly TL, Wilson KE, Heymsfield SB (2009). Dual energy X-ray absorptiometry body composition reference values from NHANES. PLoS One.

[10] Mentzel HJ, Blume J, Boettcher J, Lehmann G, Tuchscherer D, Pfeil A, Kramer A, Malich A, Kauf E, Hein G, Kaiser WA (2006). The potential of digital X-ray radiogrammetry (DXR) in the assessment of osteopenia in children with chronic inflammatory bowel disease. Pediatr Radiol.

[11] Mergler S, Evenhuis HM, Boot AM, De Man SA, Bindels-De Heus KG, Huijbers WA, Penning C (2009). Epidemiology of low bone mineral density and fractures in children with severe cerebral palsy: a systematic review. Dev Med Child Neurol.

[12] Mergler S, Rieken R, Tibboel D, Evenhuis HM, van Rijn RR, Penning C (2012). Lumbar spine and total-body dual-energy X-ray absorptiometry in children with severe neurological impairment and intellectual disability: a pilot study of artefacts and disrupting factors. Pediatr Radiol.

[13] Mergler S, de Man SA, Boot AM, KGCBB-d H, Huijbers WAR, van Rijn RR, Penning C, Evenhuis HM (2016). Automated radiogrammetry is a feasible method for measuring bone quality and bone maturation in severely disabled children. Pediatr Radiol.

[14] Messina C, Lastella G, Sorce S, Piodi LP, Rodari G, Giavoli C, Marchelli D, Guglielmi G, Ulivieri FM (2018). Pediatric dual-energy X-ray absorptiometry in clinical practice: what the clinicians need to know. Eur J Radiol.

[15] Neelis E, Rijnen N, Sluimer J, Olieman J, Rizopoulos D, Wijnen R, Rings E, de Koning B, Hulst J (2018). Bone health of children with intestinal failure measured by dual energy X-ray absorptiometry and digital X-ray radiogrammetry. Clin Nutr.

[16] Nusman CM, Anink J, Otten MH, van Rossum MA, van Rijn RR, Maas M, van Suijlekom-Smit LW (2015). Bone health of patients with juvenile idiopathic arthritis: a comparison between dual-energy X-ray absorptiometry and digital X-ray radiogrammetry. Eur J Radiol.

[17] van Rijn RR, Boot A, Wittenberg R, van der Sluis IM, van den Heuvel-Eibrink MM, Lequin MH, de MuinckKeizer-Schrama SMPF, Van Kuijk C (2006). Direct X-ray radiogrammetry versus dual-energy X-ray absorptiometry: assessment of bone density in children treated for acute lymphoblastic leukaemia and growth hormone deficiency. Pediatr Radiol.

[18] Rosholm A, Hyldstrup L, Backsgaard L, Grunkin M, Thodberg HH (2001). Estimation of bone mineral density by digital X-ray radiogrammetry: theoretical background and clinical testing. Osteoporos Int.

[19] Schundeln MM, Marschke L, Bauer JJ, Hauffa PK, Schweiger B, Fuhrer-Sakel D, Lahner H, Poeppel TD, Kiewert C, Hauffa BP, Grasemann C (2016). A piece of the puzzle: the bone health index of the BoneXpert software reflects cortical bone mineral density in pediatric and adolescent patients. PLoS One.

[20] Scott LE, Galpin JS, Glencross DK (2003). Multiple method comparison: statistical model using percentage similarity. Cytometry B Clin Cytom.

[21] Snedecor GW, Cochran WG (1980) In: Iowa State University Press (ed) Statistical methods, 7th edn, Ames

[22] Sochett EB, Makitie O (2005). Osteoporosis in chronically ill children. Ann Med.

[23] Thodberg HH (2009). Clinical review: an automated method for determination of bone age. J Clin Endocrinol Metab.

[24] Thodberg HH, van Rijn RR, Tanaka T, Martin DD, Kreiborg S (2010). A paediatric bone index derived by automated radiogrammetry. Osteoporos Int.

[25] Thodberg HH, van Rijn RR, Jenni OG, Martin DD (2017). Automated determination of bone age from hand X-rays at the end of puberty and its applicability for age estimation. Int J Legal Med.

[26] Uziel Y, Zifman E, Hashkes PJ (2009). Osteoporosis in children: pediatric and pediatric rheumatology perspective: a review. Pediatr Rheumatol Online J.

[27] van Rijn RR, Van Kuijk C (2009). Of small bones and big mistakes; bone densitometry in children revisited. Eur J Radiol.

[28] Vierucci F, Saggese G, Cimaz R (2017). Osteoporosis in childhood. Curr Opin Rheumatol.

[29] Wasserman H, O'Donnell JM, Gordon CM (2017). Use of dual energy X-ray absorptiometry in pediatric patients. Bone.

[30] Zemel BS, Kalkwarf HJ, Gilsanz V, Lappe JM, Oberfield S, Shepherd JA, Frederick MM, Huang X, Lu M, Mahboubi S, Hangartner T, Winer KK (2011). Revised reference curves for bone mineral content and areal bone mineral density according to age and sex for black and non-black children: results of the bone mineral density in childhood study. J Clin Endocrinol Metab.

